# Supplementary material for: Dietary Protein Intake and Physical Function in Māori and Non-Māori Adults of Advanced Age in New Zealand: LiLACS NZ
Source: Nutrients. 2023 Mar 29;15(7):1664. doi: 10.3390/nu15071664 (PMC10096528; doi:10.3390/nu15071664)
Supplement: Supplementary file 1 [file nutrients-15-01664-s001.zip › nutrients-2267512-supplementary.pdf]

nutrients

MDPI

Supplementary materials

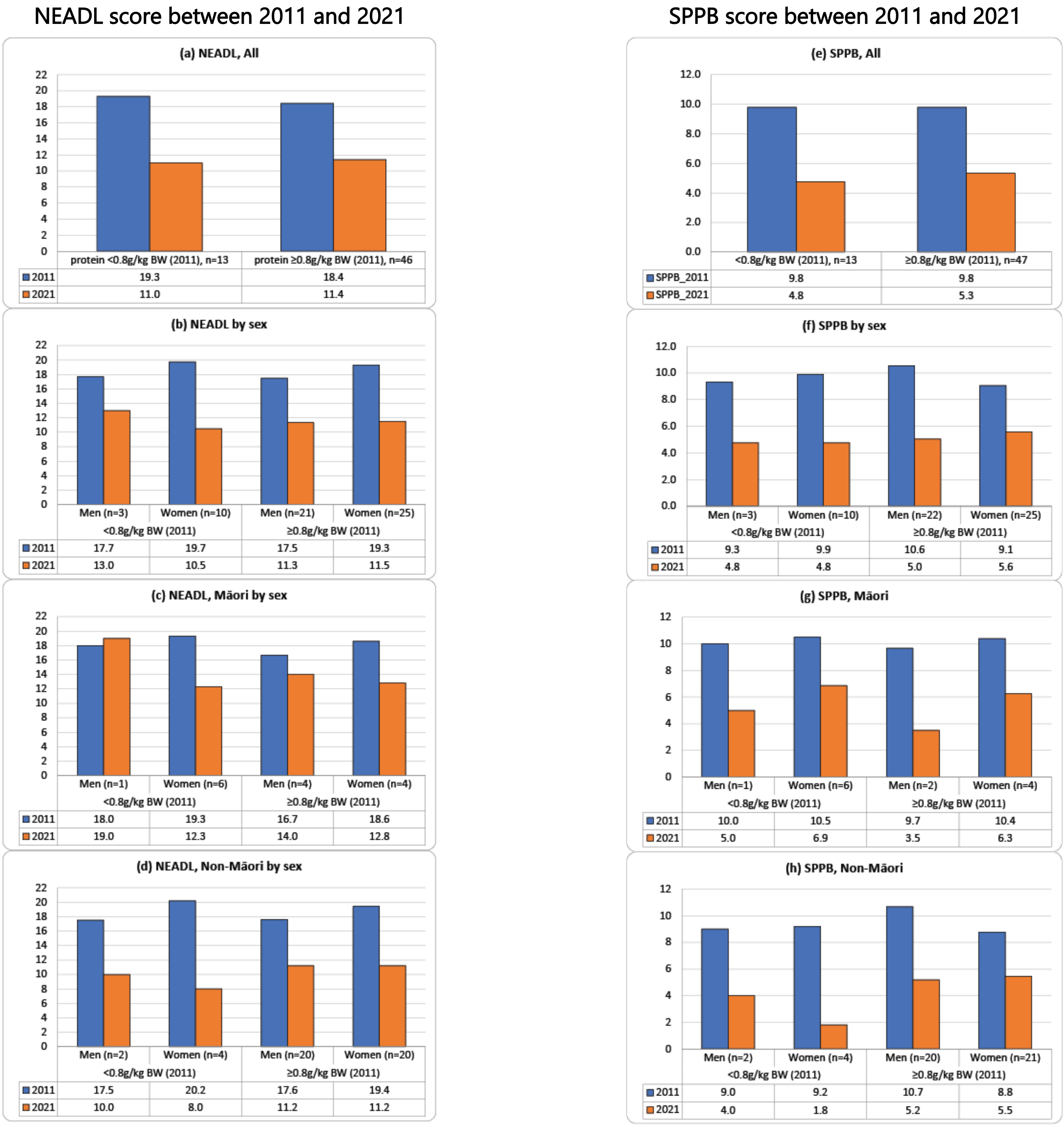


*Figure S1. Trend in 2021 NEADL and SPPB scores in Relation to Adequacy of Protein Intake in 2011*

NEADL= Nottingham Extended Activities of Daily Living, SPPB = Short Physical Performance Battery

*Note* – adequacy of protein intake is measured against meeting NZ RDI for protein ≥0.8g/kg BW/day at 2011.

(**a**) Trend in NEADL score between 2011 and 2021 based on protein intake adequacy in all participants; (**b**) Trend in NEADL score between 2011 and 2021 based on protein intake adequacy in all participants by gender; **(c)** Trend in NEADL score between 2011 and 2021 based on protein intake adequacy in Māori participants; (**d**) Trend in NEADL score between 2011 and 2021 based on protein intake adequacy in Non-Māori participants; (**e**) Trend in SPPB score between 2011 and 2021 based on protein intake adequacy in all participants; (**f**) Trend in SPPB score between 2011 and 2021 based on protein intake adequacy in all participants by gender; (**g**) Trend in SPPB score between 2011 and 2021 based on protein intake adequacy in Māori participants; (**h**) Trend in SPPB score between 2011 and 2021 based on protein intake adequacy in Non-Māori participants.

*Table S1 – GLM Models - Relationship Between 2021 NEADL Score and 2011 Protein Intake in all Participants*

| | **B (95% CI), P-value** | |
|---|---|---|
| **Variables** | **Model 1** | **Model 2** |
| Protein intake 2011 (g/kg BW/day)[#] | 1.694 (-3.732 - 7.119), 0.541 | 2.461 (-1.806 - 6.729), 0.258 |
| Age, years | | 0.501 (-0.340 - 1.341), 0.243 |
| Gender (ref: Women) | | 1.156 (-1.329 - 3.642), 0.362 |
| Ethnicity (ref: Māori) | | -3.446 (-6.818 - -0.074), 0.045* |
| NZ deprivation index - low (ref: High)[a] | | 0.679 (-2.454 - 3.812), 0.671 |
| NZ deprivation index - medium (ref: High)[a] | | -4.035 (-6.804 - -1.266), 0.004* |
| Who do you live with (ref: With others) | | 6.304 (3.859 - 8.749), <0.001* |

[a] NZ Deprivation index: Low – status 1-3, Middle – status 4-7, High – status 8-10

*Statistically significant at p<0.05

*Table S2 - GLM Models - Relationship Between 2021 SPPB Score and 2011 Protein Intake in all Participants*

| | **B (95% CI), P-Value** | |
|---|---|---|
| **Variables** | **Model 1** | **Model 2** |
| Protein intake 2011 (g/kg BW/day)[#] | 0.1808 (-2.210 - 3.827), 0.600 | 0.117 (-2.879 - 3.113), 0.939 |
| Age, years | | 0.207 (-0.416 - 0.830), 0.515 |
| Gender (ref: Women) | | -0.303 (-2.114 - 1.508), 0.743 |
| Ethnicity (ref: Māori) | | -1.758 (-4.269 - 0.752), 0.170 |
| NZ deprivation index - low (ref: High)[a] | | -0.678 (-2.946 - 1.591), 0.558 |
| NZ deprivation index - medium (ref: High)[a] | | -0.576 (-2.616 - 1.464), 0.580 |
| Who do you live with (ref: With others) | | 1.645 (-0.110 - 3.400), 0.066 |

[a] NZ Deprivation index: Low – status 1-3, Middle – status 4-7, High – status 8-10.

*Statistically significant at p<0.05
